# Compared to Fishmeal, Dietary Soybean Meal Improves the Reproductive Performance of Female Yellow Catfish (*Pelteobagrus fulvidraco*) Broodstock

**DOI:** 10.1155/2023/6240803

**Published:** 2023-04-20

**Authors:** Zheng Chen, Shuzhan Fei, Cui Liu, Yuanhui Duan, Haokun Liu, Dong Han, Junyan Jin, Yunxia Yang, Xiaoming Zhu, Shouqi Xie

**Affiliations:** ^1^State Key Laboratory of Freshwater Ecology and Biotechnology, Institute of Hydrobiology, Chinese Academy of Sciences, Wuhan 430072, China; ^2^University of Chinese Academy of Sciences, Beijing 100049, China; ^3^HAID Research Institute, Guangdong HAID Group Co., Ltd., Guangzhou 511400, China; ^4^Hubei Hongshan Laboratory, Wuhan 430070, China; ^5^The Innovative Academy of Seed Design, Chinese Academy of Sciences, Wuhan 430072, China

## Abstract

To investigate the effects of different dietary protein sources on the reproductive performance of female broodstock, yellow catfish (*Pelteobagrus fulvidraco*) were fed with three experimental diets using fishmeal (FM), soybean meal (SBM), and rapeseed meal (RSM) as main protein sources, respectively. Females (initial weight: 64.56 ± 0.45 g) were distributed into 9 net cages for feeding trial. Results indicated that 30% dietary SBM improved the reproductive performance for higher gonadosomatic index (GSI), relative fecundity, total egg production, egg diameter, and hatching rate. In addition, SBM and RSM diets resulted in higher estradiol (E2), vitellogenin (VTG), luteinizing hormones (LH), and lower follicle-stimulating hormone (FSH) and testosterone (T) in plasma (*P* < 0.05) of female broodstock. Dietary SBM and RSM also resulted in lower mesenteric fat index (MFI), plasma total cholesterol (TC), plasma total bilirubin (T-Bil) contents, and gonadal cortisol concentrations, while dietary SBM downregulated the transcription levels of steroidogenesis-related proteins by negative feedback (*P* < 0.05). The results demonstrated that dietary SBM and RSM could promote sex steroid hormone and VTG biosynthesis and showed hypocholesterolemic effects. Besides, 30% dietary SBM inclusion could improve the reproductive performance of female yellow catfish broodstock.

## 1. Introduction

Fishmeal (FM) is the most important protein ingredient used in fish diets for balanced nutrition and high digestibility [1]. However, the decline in the amount of captured fish globally and the rapid development of intensive aquaculture have contributed to its higher price [2]. With abundant resources, low prices, and high availability, using plant proteins as alternative ingredients to reduce the amount of FM in feeds is vital and significant for sustainable aquaculture. Among all plant proteins, soybean meal (SBM) and rapeseed meal (RSM) are the two most widely used protein sources for aquafeeds. According to the statistics of the China National Grain and Oils Information Center, the soybean meal and rapeseed meal domestic consumptions in China in 2021 were 75,082,000 and 13,100,000 tons, respectively.

Broodstock nutrition is crucial for gonadal maturation and egg production of the females. Food restriction and nutritional deficiency may result in impaired reproductive performance [3]. Nutrients like lipids, proteins, vitamins, and minerals have been shown to affect the fecundity of fish [4–6]. There are abundant proteins in organisms that may serve in transport, storage, or membranes as well as providing amino acids for gonadal development. There are studies being conducted to determine the effects of dietary protein levels, essential amino acids, and protein-lipid source interaction on the reproduction of broodstock [7, 8]. But few studies were designed to evaluate the effects of alternative plant proteins on reproduction [8, 9]. More attention was paid to the growth and health of fish for the unbalanced amino acid compositions and antinutritional factors in SBM and RSM. Some nutrients, like nonstarch polysaccharides, saponins in SBM, glucosinolates, tannins in RSM and protease inhibitors, and phytic acid in both of them, may have negative effects on the growth and health of fish [10, 11]. Meanwhile, some nutrients, like soy isoflavones, lecithin in SBM, and sinigrin and phenolic compounds, may be beneficial for fish health [12–15]. During the breeding season, the nutrients taken by fish not only provide nutrients and energy for their body growth but also for ovarian development. The production of good-quality eggs is closely related to maternal contributions. So it is necessary to compare the effects of different dietary protein sources on the reproductive performance of female broodstock.

As for their effects on reproductive performance, reproductive impairment caused by dietary inclusion of SBM was found in goldfish (*Carassius auratus*) [9], and Kemski et al. [16] found that nutritional programming could eliminate the negative effects of SBM-based diet on reproduction of yellow perch (*Perca flavescens*). As the first restricted amino acid in RSM, lysine could improve the fertilization rate of yellow perch [6]. Soy isoflavone like genistein showed a positive effect on growth but did not show an apparent estrogenic effect [17]. So it is necessary to determine the effects of dietary SBM and RSM inclusion on the reproduction of fish.

Yellow catfish (*Pelteobagrus fulvidraco*) is one of the popular special aquaculture species in China. As a kind of high-quality freshwater aquatic food, it possesses huge aquaculture production, which reached 587,822 tons in 2021 and still has a very potential market in China. To provide more larvae, it is effective to improve the spawning performance of females by the regulation of broodstock nutrition. Furthermore, the suitable dietary protein level for female yellow catfish was evaluated, and we found that a diet with SBM and RSM inclusion may be better for reproduction in our previous study [18]. Even though SBM and RSM are widely used in commercial feeds of yellow catfish, there are still concerns that their replacement could affect reproductive performance. So this study was designed to investigate the effects of different dietary protein sources on reproductive performance and to figure out whether endocrine disruption is being found with the inclusion of SBM and RSM in the diets.

## 2. Materials and Methods

All procedures were approved under principles for the use and care of experimental animals (IHB, CAS, approval ID: IHB 2013724) to avoid suffering.

### 2.1. Experimental Diets

Three semipurified diets using fishmeal, soybean meal, and rapeseed meal as main protein sources were formulated and were named FM, SBM, and RSM, respectively. For containing much higher protein and less residual oil, solvent-extraction SBM and RSM were used. The protein level was set at 46%, which is suitable for growth and reproduction according to our previous study [18], and casein was used as a base for all groups. Only casein and fishmeal were used as protein sources in the FM group as a control, and then, 30% SBM and 30% RSM were used to replace 19.4% and 16.7% FM, respectively (Table 1). The diets were manufactured by the HAID Research Institute (Guangdong HAID Group Co., Ltd., Guangzhou, China), and quality control checks during the grinding, mixing, extrusion, drying, coating, and cooling process were performed carefully. The extruded feeds showed stable floating ability and water durability, and their diameter was 2 mm. Amino acid profiles of all diets are presented in Table 2, and the analyzed soy isoflavone concentrations of the SBM diet are presented in Table 3. The lecithin content of all diets is lower than the lower limit of the detection (<3.6 mg/g diet).

### 2.2. Experimental Animals and Feeding Management

The feeding trial was conducted in the outdoor pond aquaculture system of the Haioudao experimental base of HAID Research Institute. Yellow catfish broodstock were obtained from the Bairong farm (Foshan, China) and domesticated under experimental conditions for two weeks. After being fasted for 24 h, 270 healthy females (initial weight: 64.56 ± 0.45 g) were equally distributed to 9 culture net cages (1.0 m × 1.0 m × 1.0 m), which were randomly divided into three treatments. Males were fed with a mixture of the three diets. It took six weeks for the feeding trial to conclude, and broodstock were fed to apparent satiation twice a day at 7 : 30 and 17 : 30. Meanwhile, feed intake was recorded every day and water quality parameters were collected once a week. Conditions were kept stable during the whole feeding trial (water temperature 27 ± 3°C; dissolved oxygen content >6.5 mg/L; total ammonia <0.1 mg/L; pH 6.9–7.6).

### 2.3. Sample Collection

At the end of the experiment, females were sampled after being fasted for 24 hours. All females in each cage were counted and weighed. Then, six females for sample collection were selected randomly and euthanized with 80 mg/L MS-222, of which two females were frozen for whole-body proximate analysis. The body length and weight of two females were collected to obtain the condition factor (CF), and then, their liver, viscera, mesenteric fat, and gonad were dissected and weighed for the hepatosomatic index (HSI), viscerosomatic index (VSI), mesenteric fat index (MFI), and gonadosomatic index (GSI). The remaining two fish were dissected after blood sampling from the caudal vein using heparinized syringes. Then, gonadal and hepatic samples were quick-frozen in liquid nitrogen and stored at -80°C for gene expression determination and lipidomics analysis. Plasma samples were obtained by centrifugation of collected blood samples (3500 rpm, 15 min, 4°C) and then stored at -80°C for analysis of biochemical indexes and hormone contents.

### 2.4. Chemical Analysis

The AOAC methods [19] were used to determine the crude protein, lipid, dry matter, and ash contents of two fish from each cage and three experimental diets. These fish and feeds were dried to a constant weight at 105°C to measure moisture contents. Then, crude lipid and protein contents were determined using a Soxtec™ 2055 (Tecator™ Technology, Foss, Hilleroed, Denmark) and a KD-310-Autoanalyzer (OPSIS, Furulund, Sweden), respectively. A muffle furnace (KS-12D-16A, Yingshan Jianli Electric Furnace Manufacturing Co., Ltd., Huangang, China) was used to measure the ash content (12 hours, 550°C). For plasma, a BS-460 automatic biochemical analyser (Shenzhen Mindray Bio-Medical Electronics Co., Ltd., Shenzhen, China) was used to collect biochemical indexes. An A300 amino acid analyser (membraPure, Hennigsdorf, Germany) was used to determine the amino acid profiles of diets, and high-performance liquid chromatography (1260 Infinity II, Agilent Technologies Inc., Santa Clara, USA) was used to determine soy isoflavone concentrations of SBM diet (GB/T 23788-2009, National Standard of the PRC) and the lecithin content of all diets (GB/T 5009.272-2016, National Standard of the PRC).

### 2.5. Artificial Reproduction

After the feeding trial, two females from each net cage and six males were randomly selected and kept in indoor tanks for breeding. Artificial reproduction was performed based on the methods of our previous study in late May [18]. The females were injected at a dose of 20 ng domperidone (DOM), 16 *μ*g luteinizing hormone-releasing hormone A2 (LHRH-A2), and 1500 IU human chorionic gonadotropin (HCG) (Ningbo Second Hormone Factory, Ningbo, China) per kg body weight. Meanwhile, the males were performed with half of the dosage. Two rounds of injection were performed for all broodstock, with only a quarter injected in the first round and the rest injected in the second ten hours later. As the females matured for spawning, five mature males weighing 100~120 g were chosen, and testes were collected after males were being anesthetized with MS-222. Sperm were obtained by a grinder and preserved in sperm preservative solution for 10 times dilution. Then, eggs were collected, and sperm were added for insemination at a ratio of 1 mL of sperm diluent to 1 kg of eggs. Each group of eggs was then placed in different labeled white porcelain trays and immersed in clean, oxygen-rich water for incubation.

### 2.6. Reproductive Performance

About 0.5 g gonadal samples of two females from each cage were collected and counted for the determination of egg weight, egg diameter, and total egg production. After artificial propagation, about 300 eggs were randomly selected from each female at the early gastrula stage (12 hours past fertilization) for calculating the fertilization rate. After hatching, the number of larvae was counted for calculating the hatching rate.

### 2.7. Determination of Plasma Hormones

Fish vitellogenin (VTG), luteinizing hormones (LH), follicle-stimulating hormone (FSH), testosterone (T), and estradiol (E2) ELISA kits (Nanjing Jiancheng Bioengineering Institute, Nanjing, China) were performed to detect the contents of VTG and hormones in plasma samples according to the instruction.

### 2.8. Total RNA Extraction, cDNA Synthesis, and Quantitative RT–PCR

TRIzol reagent (Invitrogen, Carlsbad, CA, USA) was used to extract total RNA from gonad samples. After ensuring purity and integrity, the concentration was measured, and 1 *μ*g of extracted RNA was added to the reaction system for reverse transcription using a synthesis kit (M-MLV FirstStrand, Invitrogen). RT*–*PCR was carried out on a LightCycler 480 II system (Roche, Basel, Switzerland), and Table 4 lists the primers used in the real-time PCR system for which *β-actin* and *ef1a* were selected as housekeeping genes. The PCR program was set as follows: 5 min preincubation at 95°C, 40 cycles with 10 s at 95°C, 20 s at melting temperature, and 10 s at 75°C. Based on the method of Vandesompele et al. [20], relative expression levels were calculated.

### 2.9. Hepatic Lipidomics Analysis

Liver tissues (25 mg) from the FM, SBM, and RSM groups (*n* = 6) were weighed and mixed with 800 *μ*L of extract (dichloromethane/methanol = 3 : 1, *v*/*v*, -20°C precooling) in a 1.5-milliliter Eppendorf tube. After evaluating the repeatability and stability of high-performance liquid chromatography-mass spectrometry (LC-MS) analysis, a LC-MS system consisting of ultrahigh-performance liquid phase (Waters 2D UPLC, Waters, USA) and Q Exactive high-resolution mass spectrometer (Thermo Fisher Scientific, USA) was used for the separation and detection of lipid metabolites in this experiment. The procedures and detailed parameters were in accordance with the method of Bai et al. [21].

### 2.10. Gonadal Cortisol Concentrations

Gonadal samples (50 mg, *n* = 3) from each group were quantitatively detected by a liquid-mass spectrometry system consisting of ACQUITY UPLC I-Class ultrahigh-performance liquid chromatography (Waters, USA) and QTRAP 6500 triple quadrupole mass spectrometry (SCIEX, USA).

### 2.11. Statistical Analyses

One-way ANOVA was carried out after all the data had been subjected to tests for normality and homogeneity of variance, which were performed on SPSS 25.0 (IBM, Chicago, IL, USA). Significant differences between groups were detected using Duncan's new multiple range test, and the significance level was set at *P* < 0.05. The original lipidomics data exported by the software Thermo Scientific LipidSearch [22] was imported into metaX [23] for data preprocessing and subsequent analysis. The partial least squares method-discriminant analysis (PLS-DA) model among the three groups of samples was established after the log2 logarithm transformation of the data. The screening criteria for differential lipid molecules used in this project are fold change ≥ 1.2 or ≤ 0.83, the variable importance in the projection (VIP) > 1, and *P* value of *T*-test < 0.05.

## 3. Results

### 3.1. Feed Utilization and Morphological Parameters

No significant difference was found in the growth of female yellow catfish broodstock (*P* > 0.05) (Table 5). However, the FE and CF were significantly higher in the FM group than in the RSM group, and the VSI and LDE were significantly higher in the SBM group than in the RSM group (*P* < 0.05). In addition, the FM group had significantly higher HSI than the SBM group (*P* < 0.05). Meanwhile, the FM group shows the highest MFI and the lowest BL among all groups (*P* < 0.05).

### 3.2. Body Composition and Plasma Biochemical Indexes

Effects of different protein sources on the body composition of female yellow catfish broodstock are shown in Table 6, in which the SBM group showed significantly lower moisture contents and significantly higher crude lipid contents than other groups (*P* < 0.05). However, the RSM group showed significantly lower crude lipid contents than the FM group (*P* < 0.05). As shown in Table 7, the SBM and RSM groups showed higher GLU and Ca^2+^ contents and lower TC and T-Bil contents in plasma (*P* < 0.05). In addition, the SBM group showed the highest HDL-C contents and the lowest AST and ALT contents (*P* < 0.05). No significant difference in plasma TG, LDL-C, AKP, TP, and ALB contents was found among all groups (*P* > 0.05).

### 3.3. Reproductive Performance

**Figure 1:**
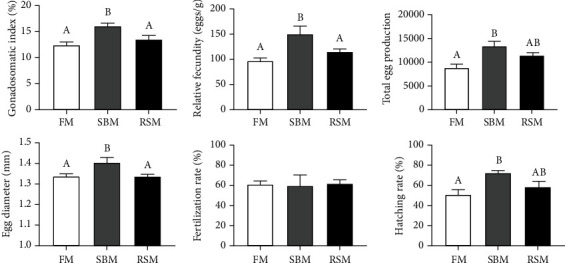
Effects of different protein sources on reproductive performance of female yellow catfish broodstock. GSI: gonadosomatic index (%) = 100 × (ovary weight)/(body weight); relative fecundity = total egg total egg production per female/mean weight of female; fertilization rate (%) = 100 × (number of fertilized eggs/total number of eggs); hatching rate (%) = 100 × (number of hatched fries/number of fertilized eggs). Values are expressed as means ± SE; different letters represent the statistically significant differences (*P* < 0.05).

As shown in Figure 1, GSI, relative fecundity, total egg production, and egg diameter were significantly higher in the SBM group than in the FM group (*P* < 0.05). Consequently, the hatching rate of the SBM group was also higher than that of the FM group (*P* < 0.05), even though no significant difference was found in fertilization rates among all groups (*P* > 0.05). Moreover, the difference in reproductive performance between the RSM group and the FM group was not significant (*P* > 0.05).

### 3.4. Plasma Hormone Contents and Vitellogenin (VTG) Contents

**Figure 2:**
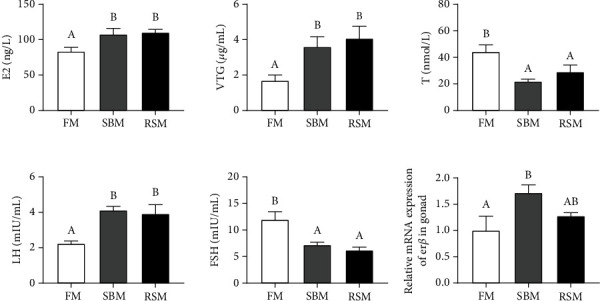
Plasma hormone contents, VTG contents, and relative mRNA expression of gonadal *erβ* of female yellow catfish broodstock fed experimental diets. E2: estradiol; VTG: vitellogenin; T: testosterone; LH: luteinizing hormone; FSH: follicle stimulating hormone. Values are expressed as means ± SE; different letters represent the statistically significant differences (*P* < 0.05).

Plasma hormone contents, VTG contents, and relative mRNA expression of gonadal *erβ* of female yellow catfish broodstock fed experimental diets are shown in Figure 2. The SBM and RSM groups showed significantly higher E2, VTG, and LH contents and significantly lower T and FSH contents than the FM group in plasma (*P* < 0.05). We also found that the relative mRNA expression of gonadal *erβ* was significantly upregulated in the SBM group than in the FM group (*P* < 0.05). SBM and RSM showed similar effects on plasma hormone and VTG contents.

### 3.5. Gonadal mRNA Levels of Steroidogenesis-Related Genes

**Figure 3:**
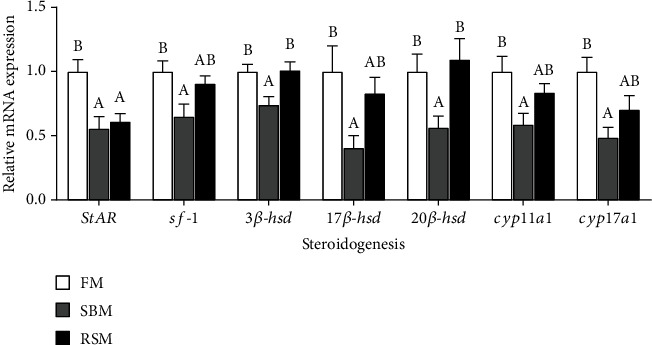
Effect of different dietary protein sources on gonadal mRNA expressions of steroidogenesis-related genes of female yellow catfish broodstock. Values are expressed as means ± SE; different letters represent the statistically significant differences (*P* < 0.05).

The gonadal mRNA expression levels of *StAR*, *sf-1*, *3β-hsd*, *17β-hsd*, *20β-hsd*, *cyp11a1*, and *cyp17a1* were significantly downregulated in the SBM group than in the FM group (*P* < 0.05) (Figure 3). RSM diet only downregulated the mRNA expression levels of *StAR*, and other genes involved in the steroidogenesis of female yellow catfish broodstock were not significantly affected (*P* > 0.05).

### 3.6. Hepatic Lipidomics

**Figure 4:**
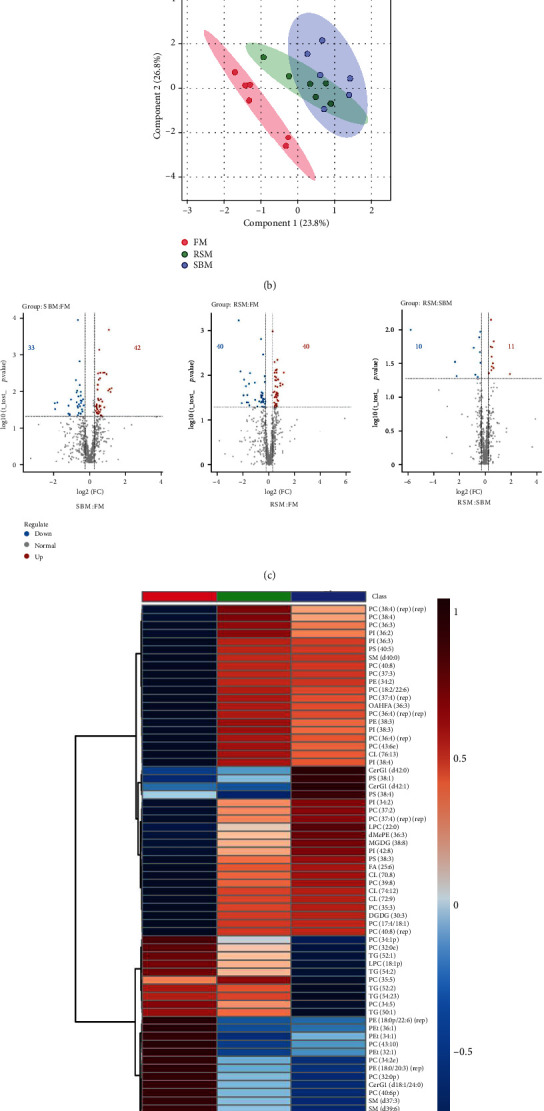
Effect of different dietary protein sources on hepatic lipid metabolites of female yellow catfish broodstock based on the lipidomics approach. (a). Analysis of mean peak intensity of main class lipid molecules; values are expressed as means ± SE (*n* = 6); different letters represent the statistically significant differences (*P* < 0.05). (b). PLS-DA discriminant analysis model score map. (c). Volcano plots of all identified lipid metabolites from lipidomics; different colors were used to represent downregulated (blue), upregulated (red), or nonsignificant (gray) metabolites.(d). Heatmap of differential subclass lipid molecules. *Z* score normalized ionic strength for each metabolite was represented by different colors: high (red), low (blue), or average (white).

The differences in hepatic lipid metabolites among all groups are presented in Figure 4. A total of 744 lipid metabolites in 33 subclasses were identified. As shown in Figure 4(a), the SBM and RSM diet increased the DG (diglyceride), FA (fatty acid), DGDG (digalactosyldiacylglycerol), and CL (cardiolipin) contents but decreased the ChE (cholesteryl ester) contents in the liver significantly (*P* < 0.05). And TG (triglyceride) contents were significantly higher in the RSM group than in the SBM group (*P* < 0.05). PLS-DA discriminant analysis model score map was performed to investigate comparative interpretations and variation among groups. The FM group was clearly distinguished from the SBM and RSM groups, but the SBM group and the RSM group overlapped each other (Figure 4(b)), suggesting the differences of the effects of animal protein source (FM) and plant protein sources (SBM and RSM) on hepatic lipids and the similarity of the effects of SBM and RSM on hepatic lipids. Likewise, volcano plot analysis of RSM:SBM showed 10 lipid metabolites downregulated and 11 lipid metabolites upregulated, and fewer lipids were distinguished between RSM and SBM than SBM vs. FM and RSM vs. FM. For SBM vs. FM, there were 33 lipids downregulated and 42 lipids upregulated (Figure 4(c)). In addition, the differences in the levels of these distinguished lipids are presented in Figure 4(d). These lipids may be related to the improvement of the reproductive performance of female yellow catfish. The hepatic contents of fatty acids including OAHFA 36 : 3 ((O-acyl)-1-hydroxy fatty acid) and FA 25 : 6 were upregulated by dietary SBM. And the contents of five PIs (phosphatidylinositol, PI 36 : 2, PI 36 : 3, PI 38 : 3, PI 34 : 2, and PI 42 : 8), four CLs (CL 76 : 13, CL 70 : 8, CL 74 : 12, and CL 72 : 9), four PSs (phosphatidylserine, PS 40 : 5, PS 38 : 1, PS 38 : 3, and PS 38 : 4), MGDG 38 : 8 (monogalactosyldiacylglycerol), DGDG 30 : 3, and dMePE 36 : 3 (dimethylphosphatidylethanolamine) in the SBM group were also enhanced, but the contents of four TGs (TG 52 : 1, TG 52 : 2, TG 54 : 3, and TG 50 : 1) and PEts (phosphatidylethanol, PEt 36 : 1, PEt 34 : 1, and PEt 32 : 1) were higher in the FM group than in the SBM group. Other lipids like PCs and PEs were partly increased and partly decreased.

### 3.7. Gonadal Cortisol Concentrations

**Figure 5:**
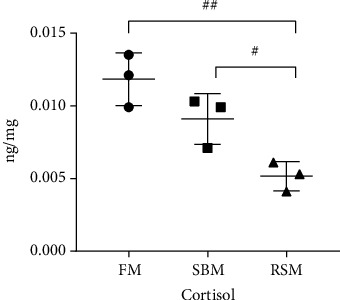
Effect of different dietary protein sources on gonadal cortisol concentrations of female yellow catfish broodstock. Values are expressed as means ± SE (*n* = 3); ^#^ represents the statistically significant differences (*P* < 0.05), and ^##^ represents the extremely significant differences (*P* < 0.01).

As presented in Figure 5, the gonadal cortisol concentrations were significantly lower in the RSM group than in the SBM group (*P* < 0.05) and extremely significantly lower in the RSM group than in the FM group (*P* < 0.01).

## 4. Discussion

The present study revealed that 30% inclusion of SBM and RSM in the diets did not suppress the growth of female yellow catfish broodstock. For gilthead sea bream (*Sparus aurata*), higher SBM inclusion level to 42.6% in the diet also did not result in depressed growth performance [24]. Previous study showed that even 75% and 80% FM protein replaced by SBM protein did not influence the growth of tilapia [25] and Florida pompano (*Trachinotus carolinus*, *L.*) [26], respectively. But using RSM as an alternative protein source is not as suitable as SBM. The inclusion of RSM in diets should be lower than 15% for tilapia [27], and increasing the substitute level to 30% resulted in the growth inhibition of *Pseudobagrus ussuriensis* [28]. Even though no significant decrease in FBW, SGR, and FR was observed in our study, 30% inclusion of RSM resulted in significantly lower FE and CF.

The quality of dietary protein can not only have an impact on feed utilization but also reproductive performance, since adequate intake of essential nutrients supports growth, metabolism, and gonadal development [29, 30]. Broodstock nutrition may promote the production of high-quality eggs by assembling maternal nutrients into the oocyte in the ovary. Except for the gonadosomatic index, reproductive performance was also evaluated based on fecundity, egg size, gonadotropin and sex hormone production, fertilization rate, hatching rate, and so on. In the present study, the reproductive performance seems to be improved by 30% inclusion of SBM in the broodstock diet for significantly higher gonadosomatic index, relative fecundity, total egg production, egg diameter, and hatching rate observed in the SBM group compared to the FM group. As we know, there are soy isoflavones in the SBM diet and these are phytoestrogens, which showed a similar structure to estrogen. And dietary soy isoflavones was reported to improve the reproduction of sows [31]. However, decreased fertilization and hatching rate caused by SBM were found on goldfish, and SBM inclusion higher than 35% impaired the reproduction for sex hormone biosynthesis disruption [9]. Due to the dose-dependent effect of soy isoflavones, positive effects are observed at low contents, and negative effects are observed at high contents. The difference may be attributed to lower SBM inclusion and thus lower soy isoflavones content in the present study. In addition to the estrogenic effect to induce the synthesis of VTG [32], soy isoflavones may improve reproduction by improving adipose inflammation, modulating lipid metabolism, and enhancing the antioxidation [33–35].

Interestingly, dietary SBM also could regulate the endocrine system of reproduction. Plasma concentrations of E2 and VTG increased significantly in the SBM group, and it was in accordance with the results on female gibel carp, of which soy isoflavone increased the serum E2 and VTG levels [36]. Hypothalamo-pituitary-gonad (HPG) axis plays an important role in reproduction for stimulating the synthesis of sex steroid hormones and their relevant feedback regulation. During the maturation of gonads, T would be aromatized and converted to E2, which would be released into the blood and stimulates the liver to synthesize VTG.

As the precursor of egg yolk, VTG could transport protein and some lipids from the liver through the blood to the growing oocytes in the ovary [37]. Then, nutrients are transported into eggs and provide energy and nutrients for embryo development. In the present study, dietary SBM and RSM promoted the secretion of E2 and VTG, but only SBM promoted gonadal maturation and increased the egg production and egg size of female yellow catfish. It is speculated that female broodstock in the RSM group failed to promote the incorporation of VTG into oocytes. It is generally believed that the quality of larger eggs is better for more nutrient reservation. Larger eggs in the SBM group also represented a higher hatching rate. In addition, E2 could regulate the intestinal calcium absorption and increase blood calcium levels [38]. Dietary SBM and RSM resulted in higher plasma calcium level and longer body length. Calcium is essential for the growth of bones, so dietary SBM and RSM may contribute to the growth of fish body length by estrogenic activities.

Dietary SBM and RSM promoted the secretion of E2. In return, the steroid hormone may regulate the release of gonadotropins, including LH and FSH, through feedback effect. However, plasma LH contents were increased by dietary SBM and RSM, while plasma FSH contents were decreased by dietary SBM and RSM. This may be due to their different functions and biological activities. FSH is usually secreted in large quantities at the early stage of gonadal development and plays a major role in stimulating the secretion of E2 and T in the gonad. But LH is secreted in large quantities in the late gonadal development and the maturity stage and stimulates the production of steroid hormones that induce oocytes to mature at last. Steroidogenic enzymes are vital for the biosynthesis of E2, and sf-1 is an important transcription factor in steroidogenesis [39]. Besides, StAR can mediate the transfer of cholesterol from the outer to the inner mitochondrial membrane and has an important rate-limiting effect on steroid hormone synthesis [40]. The gonadal maturity of the SBM group was higher than other groups, so the gene expression of steroidogenesis-related proteins may be downregulated by negative feedback. And similar feedback regulation in mature ovaries was found on the tongue sole (*Cynoglossus semilaevis*) [41].

Lipids are essential for gametogenesis and gonadal maturation. The lowest crude lipid content of female yellow catfish was observed in the RSM group, possibly indicating that RSM affects the absorption and utilization of dietary lipids. With more and larger eggs in the gonads, the whole-body crude lipid content was higher in the SBM group than in other groups. Thus, VSI and LDE were higher in the SBM group than in the RSM group. Furthermore, the dietary SBM and RSM resulted in lower MFI. The FM diet may provide excess nutrients, which are deposited in the fish as mesenteric fat. Excessive lipid accumulation usually impairs female reproductive performance [42]. Consequently, plasma TC and T-Bil contents were increased by the FM diet. Even though no difference was found on plasma TG contents, HDL-C contents were elevated by dietary SBM inclusion. For HDL-C could transport lipids from the liver to the ovary, essential fatty acids obtained from diets promote gonadal development. As for lipid metabolism in the liver, the SBM and RSM diets showed similar effects, but the FM diet showed a different pattern. Decreased DGs, FAs, DGDGs, and CLs and increased ChEs were observed in the FM group which suggested lipid metabolism disorders caused by excessive lipids. The differences on hepatic lipid metabolites of all groups may be related to steroid synthesis or gonadal maturation. Even though PCs are a major component of egg yolk and can be easily obtained from soybeans, the change in PC content did not show any pattern. Some PCs were upregulated and some were downregulated by dietary SBM. Vitellogenin has a high affinity for liposomes containing PS (phosphatidylserine) [43]. The content of serine is the highest among all amino acids in lipovitellin and existed in the form of PS. As a precursor of PI3K (phosphatidylinositol 3-kinase), PI could regulate growth and metabolism and promote estrogen receptor transcriptional activity [44, 45]. High contents of PIs and PSs in the SBM group may promote the gonadal maturation and the secretion of E2.

Cortisol accumulates easily in the growing oocyte, and it has a negative effect on the surrounding follicles [46]. Higher gonadal cortisol contents in the FM group may be the inducement of impaired reproductive function. With low gonadal cortisol contents and high plasma E2 contents, no positive effects on gonadal development were brought by dietary RSM inclusion. This may be due to antinutritional factors or unbalanced amino acid profile.

In conclusion, the present study revealed that dietary SBM inclusion could improve the reproductive performance of female yellow catfish broodstock. And dietary SBM and RSM could promote the secretion of E2 and VTG and showed hypocholesterolemic effects. Hence, FM can be partially replaced with 30% SBM in the broodstock diet. But further research should be conducted to determine the effects of dietary soy isoflavones on reproduction and estrogenic activity in female yellow catfish.

## Figures and Tables

**Table 1:** Formulation and proximate composition of the experimental diets (% dry matter).

	FM	SBM	RSM
*Ingredients*			
Casein	16.20	16.20	16.20
White fishmeal	44.90	25.50	28.20
Soybean meal	0.00	30.00	0.00
Rapeseed meal	0.00	0.00	30.00
Corn starch	24.20	10.30	10.00
Fish oil	3.03	3.67	3.50
Soybean oil	3.03	3.67	3.50
Vitamin premix^1^	0.39	0.39	0.39
Mineral premix^2^	5.00	5.00	5.00
Choline chloride	0.11	0.11	0.11
Ca(H_2_PO_4_)_2_•H_2_O	1.00	1.00	1.00
Ethoxyquinoline	0.10	0.10	0.10
Cellulose	0.04	2.06	0.00
Carboxymethylcellulose sodium	2.00	2.00	2.00
*Proximate composition*			
Crude protein	46.39	46.61	46.31
Crude lipid	8.32	8.52	8.25
Moisture	7.52	7.00	7.34
Ash	9.00	8.45	8.85

^1^Vitamin premix (mg kg^−1^ diet): vitamin B_1_, 20; vitamin B_2_, 20; vitamin B_6_, 20; vitamin B_12_, 0.02; folic acid, 5; calcium patothenate, 50; inositol, 100; niacin, 100; biotin, 0.1; cellulose, 3411.88; ascorbic acid, 100; vitamin A, 11; vitamin D, 2; vitamin E, 50; vitamin K, 10; 2. Mineral premix (mg kg^−1^ diet): NaCl, 500.0; MgSO_4_·7H_2_O, 8155.6; NaH_2_PO_4_·2H_2_O, 12500.0; KH_2_PO_4_, 16000.0; Ca(H_2_PO_4_) 2H_2_O, 7650.6; FeSO_4_·7H_2_O, 2286.2; C_6_H_10_CaO_6_·5H_2_O, 1750.0; ZnSO_4_·7H_2_O, 178.0; MnSO_4_·H_2_O, 61.4; CuSO_4_·5H_2_O, 15.5; CoSO_4_·7H_2_O, 0.90; KI, 1.5; Na_2_SeO_3_, 0.60; corn starch, 899.7.

**Table 2:** Amino acid composition of the experimental diets (% dry matter).

Item	FM	SBM	RSM
Met	1.18	1.01	1.09
Lys	3.16	3.14	3.19
Thr	1.90	1.95	2.01
Ile	1.79	1.85	1.87
His	1.14	1.16	1.17
Val	2.04	2.15	2.22
Leu	3.34	3.45	3.44
Arg	1.83	1.91	1.92
Phe	1.87	2.06	1.95
Asp	3.49	3.81	3.59
Ser	1.54	1.68	1.66
Glu	5.89	6.68	6.72
Gly	1.83	1.82	1.93
Ala	2.37	2.28	2.29
(Cys)_2_	0.08	0.07	0.12
Pro	2.05	2.10	2.21
Tyr	1.40	1.40	1.42

**Table 3:** Analyzed soy isoflavone concentrations of the SBM diet (% dry matter).

Item (mg/kg)	SBM
Total daidzin	84.8
Total glycitin	20.2
Total genistin	128.0
Total daidzein	21.0
Total glycitein	24.2
Total genistein	25.8
Total isoflavones	304.0

**Table 4:** Primers used in quantitative real-time PCR analysis.

Gene	GeneBank accession no.	Forward primer (5′-3′)	Revers primer (5′-3′)
*β-Actin*	XM027148463.1	TTGGCAATGAGAGGTTCAGGT	TGCCAGGGTACATGGTGGTA
*ef1a*	XM027175544.2	TTAAAGGTGGTGGGGCCTTG	CTGTGGCAACAGGTGCAGAT
*Sf-1*	XM027148774.2	GTGCGTGTGTCTGTTTGGAC	CTGACCCTTTACTTTGCGCC
*erβ*	KT832703.1	GCCTCTCAGTCCGTCGTTCA	GTGCTAGGTGGCTTGCTGTC
*StAR*	KP707128.1	GCAGACGATCCCAACAAGAC	CCGTTGCCTCCATTCTGATG
*3β-hsd*	KP701031	AGTTCCACAAAACACCGAGC	TACACTTCACCTCCGAGTCG
*17β-hsd*	XM027161133.2	GAAAAGCATCTCACCTCCCG	AGCCAGTGATCAGAACGACC
*20β-hsd*	FJ465136.1	GTGTCGTGCTCTGCTCCCA	TTCCGCTCCTTCCTCTGT
*cyp11a1*	KP135444.1	ATGGTGAACGTCTGGGGCT	TCGTAACATTGAAGGGGGG
*cyp17a1*	HQ83264.3	TACCTGGAAGCCACAATC	TCATTCAGGAAACGCTCT

Abbreviations: *β-actin*: beta-actin; *ef1α*: elongation factor 1-alpha; *sf-1*: steroidogenic factor 1; *erβ*: estrogen receptor beta; *StAR*: steroidogenic acute regulatory protein; *3β-hsd*: 3beta-hydroxysteroid dehydrogenase; *17β-hsd*: 17beta-hydroxysteroid dehydrogenase; *20β-hsd*: 20beta-hydroxysteroid dehydrogenase; *cyp11a1*: cytochrome P450 11a1; *cyp17a1*: cytochrome P450 17a1.

**Table 5:** Feed utilization and morphological parameters of female yellow catfish broodstock fed the experimental diets.

	FM	SBM	RSM
IBW (g)	64.56 ± 0.14	64.55 ± 0.06	64.56 ± 0.09
FBW (g)	82.91 ± 1.6	83.49 ± 1.03	82.51 ± 2.24
FR (%BW/d)	1.00 ± 0.02	1.05 ± 0.02	1.08 ± 0.05
SGR (%/d)	0.59 ± 0.04	0.61 ± 0.03	0.59 ± 0.07
FE (%)	59.12 ± 2.93^b^	57.93 ± 1.89^ab^	53.21 ± 3.69^a^
PRE (%)	21.06 ± 1.05	19.91 ± 0.57	18.86 ± 1.48
LDE (%)	114.8 ± 7.70^ab^	130.57 ± 6.26^b^	94.23 ± 5.23^a^
BL (mm)	152.38 ± 2.10^a^	160.33 ± 2.20^b^	159.78 ± 2.22^b^
CF (g/cm^3^)	2.58 ± 0.07^b^	2.43 ± 0.06^ab^	2.34 ± 0.04^a^
HSI (%)	1.94 ± 0.09^b^	1.65 ± 0.07^a^	1.78 ± 0.04^ab^
VSI (%)	28.81 ± 0.83^ab^	30.94 ± 0.82^b^	27.61 ± 0.60^a^
MFI (%)	9.66 ± 0.41^b^	7.20 ± 0.55^a^	6.39 ± 0.50^a^

Values are expressed as means ± SE; different letters represent the statistically significant differences (*P* < 0.05). IBW: initial mean body weight (g); FBW: final mean body weight (g); FR: feeding rate (%BW/d) = 100 × (feed intake in dry matter)/(days × (initial body weight + final body weight)/2); SGR: specific growth rate (%/d) = 100 × [ln (final body weight) − ln (initial body weight)]/days; FE: feed efficiency (%) = 100 × (final body weight − initial body weight)/feed intake in dry matter; PRE: protein retention efficiency (%) = 100 × retained protein/protein intake; LDE: lipid deposition efficiency (%) = 100 × lipid retained in fish body/lipid intake; BL: body length (mm); CF: condition factor (g/cm^3^) = 100 × (body weight)/(body length)^3^; HSI: hepaticsomatic index (%) = 100 × (liver weight)/(whole body weight); VSI: viscerosomatic index (%) = 100 × (viscera weight)/(whole body weight); MFI: mesenteric fat index (%) = 100 × (weight of mesenteric fat)/(body weight).

**Table 6:** Effects of different protein sources on body composition of female yellow catfish broodstock.

	FM	SBM	RSM
Moisture (%)	61.88 ± 0.68^b^	60.08 ± 1.03^a^	63.14 ± 0.33^b^
Crude lipid (%)	16.18 ± 0.94^b^	17.92 ± 0.83^c^	14.65 ± 0.43^a^
Crude protein (%)	16.18 ± 0.46	16.03 ± 0.13	16.39 ± 0.17
Ash (%)	3.92 ± 0.13	3.88 ± 0.14	3.95 ± 0.08

Values are expressed as means ± SE (*n* = 3); different letters represent the statistically significant differences (*P* < 0.05).

**Table 7:** Plasma biochemical indexes of female yellow catfish broodstock fed formulated diets with different protein sources.

	FM	SBM	RSM
GLU (mmol/L)	6.32 ± 0.45^a^	8.16 ± 0.58^b^	8.03 ± 0.49^b^
TG (mmol/L)	9.86 ± 1.10	9.55 ± 0.92	10.58 ± 1.10
LDL-C (mmol/L)	2.05 ± 0.25	1.76 ± 0.17	2.29 ± 0.18
HDL-C (mmol/L)	2.11 ± 0.10^a^	2.54 ± 0.07^b^	2.29 ± 0.14^ab^
TC (mmol/L)	8.15 ± 0.51^b^	6.31 ± 0.36^a^	6.56 ± 0.31^a^
T-Bil (*μ*mol/L)	9.13 ± 0.78^b^	5.97 ± 0.83^a^	6.45 ± 0.88^a^
Ca^2+^ (mmol/L)	1.00 ± 0.15^a^	1.48 ± 0.11^b^	1.49 ± 0.05^b^
ALT (U/L)	14.85 ± 1.36^ab^	10.98 ± 0.99^a^	16.32 ± 1.72^b^
AST (U/L)	69.12 ± 5.33^b^	53.84 ± 3.53^a^	70.97 ± 2.93^b^
AKP (U/L)	35.10 ± 2.38	31.30 ± 2.75	31.33 ± 3.13
TP (g/L)	36.17 ± 1.21	39.11 ± 0.92	35.80 ± 1.45
ALB (g/L)	10.54 ± 0.32	10.31 ± 0.31	10.94 ± 0.25

Values are expressed as means ± SE (*n* = 6); different letters represent the statistically significant differences (*P* < 0.05). GLU: glucose; TG: triglyceride; LDL-C: low-density lipoprotein cholesterol; HDL-C: high-density lipoprotein cholesterol; TC: total cholesterol; T-Bil: total bilirubin; ALT: alanine aminotransferase; AST: aspartate aminotransferase. AKP: alkaline phosphatase; TP: total protein; ALB: albumin.

## Data Availability

Data are available on request.
